# Peptide-Based Rapid and Selective Detection of Mercury in Aqueous Samples with Micro-Volume Glass Capillary Fluorometer

**DOI:** 10.3390/bios14110530

**Published:** 2024-11-01

**Authors:** Marta Sosnowska, Emil Pitula, Monika Janik, Piotr Bruździak, Mateusz Śmietana, Marcin Olszewski, Dawid Nidzworski, Beata Gromadzka

**Affiliations:** 1 Department of Analysis and Chemical Synthesis, Institute of Biotechnology and Molecular Medicine, 80-180 Gdansk, Poland; m.sosnowska@ibmm.pl; 2 Institute of Microelectronics and Optoelectronics, Warsaw University of Technology, Koszykowa 75, 00-662 Warsaw, Poland; emil.pitula@pw.edu.pl (E.P.); monika.janik@pw.edu.pl (M.J.); mateusz.smietana@pw.edu.pl (M.Ś.); 3 Department of Physical Chemistry, Gdańsk University of Technology, 80-233 Gdansk, Poland; piotr.bruzdziak@pg.edu.pl; 4 Department of Glass, Institute of Microelectronics and Photonics, Łukasiewicz Research Network, Al. Lotników 32/46, 02-668 Warsaw, Poland; 5 Chair of Drug and Cosmetics Biotechnology, Faculty of Chemistry, Warsaw University of Technology, 00-664 Warsaw, Poland; marcin.olszewski@pw.edu.pl; 6 Department of In Vitro Studies, Institute of Biotechnology and Molecular Medicine, 80-180 Gdansk, Poland; dawid.nidzworski@gmail.com; 7 NanoExpo®, 80-822 Gdansk, Poland

**Keywords:** low-volume measurements, optical sensing, micro-volume glass capillary fluorometer peptide-based sensor, mercury detection, turn-off fluorescence, mercury ions (Hg^2+^) detection

## Abstract

Mercury, a toxic heavy metal produced through both natural and anthropogenic processes, is found in all of Earth’s major systems. Mercury’s bioaccumulation characteristics in the human body have a significant impact on the liver, kidneys, brain, and muscles. In order to detect Hg^2+^ ions, a highly sensitive and specific fluorescent biosensor has been developed using a novel, modified seven amino acid peptide, FY7. The tyrosine ring in the FY7 peptide sequence forms a 2:1 complex with Hg^2+^ ions that are present in the water-based sample. As a result, the peptide’s fluorescence emission decreases with higher concentrations of Hg^2+^. The FY7 peptide’s performance was tested in the presence of Hg^2+^ ions and other metal ions, revealing its sensitivity and stability despite high concentrations. Conformational changes to the FY7 structure were confirmed by FTIR studies. Simultaneously, we designed a miniaturized setup to support an in-house-developed micro-volume capillary container for volume fluorometry measurements. We compared and verified the results from the micro-volume system with those from the commercial setup. The micro-volume capillary system accommodated only 2.9 µL of sample volume, allowing for rapid, sensitive, and selective detection of toxic mercury (II) ions as low as 0.02 µM.

## 1. Introduction

Mercury is present in the environment in a variety of forms, including elemental mercury and as a component of inorganic or organic compounds. Although elemental Hg(0) is not particularly toxic, it is easily oxidized to Hg^2+^, which is both highly reactive and highly toxic [1,2,3]. Despite stringent guidelines and regulations, mercury is released to the environment through various industrial processes and uncontrolled waste disposal [4]. Exposure to mercury, primarily through ingestion and inhalation, remains a persistent concern. By consuming contaminated food or water, living organisms accumulate mercury in their tissues over time. Difficulty in eliminating inorganic Hg compounds further strengthens this accumulation, leading to dangerously elevated mercury levels. Bioaccumulated mercury ions hinder the functionality of proteins and enzymes by binding to their active sites or essential cofactors, such as thiol groups [5,6]. As a result, metabolic pathways and cellular functions are disrupted. Prolonged mercury exposure may weaken the immune system, making organisms more susceptible to infections and diseases [7,8]. Additionally, mercury generates reactive oxygen species (ROS) [9,10], which induces oxidative damage to lipids, proteins, and DNA. Mercury exposure is also associated with cardiovascular diseases, including heart attacks and epilepsy, neurological diseases like Parkinson’s and Alzheimer’s [11,12,13], as well as vision loss and potential fatality [14,15]. Furthermore, mercury can impair reproductive health, leading to developmental abnormalities, adverse effects on offspring, and reduced fertility [16,17]. Mercury is an environmentally persistent substance that resists degradation and remains in ecosystems for extended periods as it continuously cycles through air, water, and soil. The toxicity varies with dosage and exposure time; therefore, the development of new, rapid, and precise detection methods is crucial to mitigate any health risks. Current detection methods of Hg^2+^ are atomic absorption spectrometry (AAS), inductively coupled plasma (ICP), and atomic fluorescence spectrometry (AFS) [18,19,20]. However, these conventional methods are often unsuitable for routine analysis because they require a significant time investment, complex sample preparation, and specific, bulky instruments. It is essential to develop effective methods for the rapid, highly selective, and appropriate preliminary screening and field analysis of Hg^2+^. Among optical detection techniques used in general for heavy metal ions, it is essential to underscore quick and simple volume fluorometry methods. Fluorometry techniques significantly shorten the time necessary for measurements, producing nearly real-time results [21]. A variety of fluorometry techniques have been successfully employed to detect extremely low concentrations of heavy metal ions with the use of specific fluorescent probes [22,23,24,25,26]. These days, sensors that incorporate peptide motifs are starting to show promise as an alternative due to their simplicity in synthesis through 9-fluorenylmethoxycarbonyl (Fmoc) solid-phase peptide synthesis (SPPS) [27,28] and ability to be selective for metal ions.

This work proposes a new, custom designed fluorescent probe, FITC labelled FY7 peptide, meant for the sensitive and rapid detection of Hg^2+^ ions in water samples. Fluorescein isothiocyanate (FITC) was chosen as the fluorescent moiety for FY7 labeling due to its rapid reactivity with primary amines and high quantum efficiency. FITC’s isothiocyanate group forms a stable thiourea bond with amine groups, allowing for efficient and stable conjugation. Additionally, its high quantum yield ensures strong fluorescence, making it an excellent choice for sensitive detection and imaging applications. [29,30]. FITC typically exhibits strong fluorescence around 520 nm when excited at an appropriate wavelength, usually around 490 nm [31]. The FY7 peptide is designed to generate changes in its fluorescence response when it binds to Hg^2+^ ions. In water-based samples, this response can be employed to detect and quantify the presence of Hg^2+^ ions. FY7 peptide’s behavior was investigated to evaluate its potential for bio-sensing applications and environmental monitoring. Peptide’s fluorescence response and selectivity were measured when exposed to a series of low concentrations of Hg^2+^ ions, as well as in the presence of seven other, different metal ions, using a commercial microplate spectrofluorometer. The proposed FY7 sensor exhibited exceptional water solubility, selectivity, and sensitivity. As a low-cost alternative to professional setups, we designed a custom, simple volume fluorometry setup employing a glass capillary as a container. Capillaries have been used in numerous studies and present a viable and straightforward alternative to well-established containers, such as well-plates or cuvettes, but offer a much lower sample volume for single measurement [32,33,34]. Here, FY7 peptide was investigated with a capillary that required only 2.9 µL of sample volume. Comparing the results between high-end fluorometry devices and custom setups, almost identical responses were observed, highlighting the robustness of synthesized FY7 peptide. Application of the FY7 peptide in a cost-efficient, miniaturized optical setup showcases a promising advancement in fluorescence measurement technology to date.

## 2. Materials and Methods

### 2.1. Materials and Reagents

Fmoc-Rink Amide AM Resin (0.7 mmol/g) was purchased from Iris Biotech GmbH (Marktredwitz, Germany). Fmoc protected amino acids, Fmoc-βAla-OH Fmoc-Tyr(tBu)-OH, Fmoc-Lys(Boc)-OH, Fmoc-Ala-OH, Fmoc-Ser(tBu)-OH, Fmoc-Leu-OH, Fmoc-Leu-OH and Fmoc-Thr(tBu)-OH were purchased from CSBio (Shanghai) Ltd. (Shanghai, China). Fluosescein-5-isothiocyanate FITC Isomer I (FITC), Oxyma pure, N,N’-Diisopropylcarbodiimide (DIC), N,N-diisopropylethylamine (DIEA), Piperidine, 99%, extra pure, Triisopropylsilane (TIS), 4-Morpholineethanesulfonic acid sodium salt (MES), Pb(NO_3_)_2_, and MnCl_2_ were obtained from Sigma-Aldrich (St. Louis, MO, USA). N,N-Dimethylformamide (DMF), 99.8% and Trifluoroacetic Acid (TFA) for synthesis, Chromium standard solution, Cadmium standard solution, Hg(NO_3_)_2_, ZnSO_4_, NaCl, and KCl were obtained from VWR International, LLC (Randor, PA, USA). Ethyl dieter was collected from POCH S.A. (Gliwice, Poland).

D_2_O, 99.8% was purchased from Thermo Scientific Chemicals (Waltham, MA, USA). The Acetonitrile HPLC gradient grade, Acetonitrile hypergrade for LC-MS LiChrosolv^®^, water (LC-MS LiChrosolv^®^), trifluoracetic acid for the HPLC and formic acid (FA) (LC-MS LiChropur™) were purchased from Sigma-Aldrich. Double-distilled water (Hydrolab-Reference purified) with conductivity not exceeding 0.05 µS/cm was used. All other chemicals used were of analytical reagent grade unless otherwise noted.

### 2.2. Peptide Synthesis and Characterization

The FY7 peptide with sequence of FITC-βAla-Tyr-Lys-Ala-Ser-Leu-Ile-Thr-NH_2_ was synthesized on Rink Amide resin (0.1 mmol) by microwave-assisted Fmoc solid-phase peptide synthesis (SPPS) [35]. Peptide chain elongation was carried out using an Initiator+ Alstra™ (Biotage, Uppsala, Sweden) automated microwave peptide synthesizer. Couplings were performed twice for 5 min at 75 °C using Fmoc-amino acid (5 equiv.), DIC (5 equiv.), and Oxyma (5 equiv.) in DMF. The Fmoc group was removed using a 20% piperidine solution in DMF at room temperature (1 × 3 min., 1 × 10 min.). FITC was introduced at the N-terminal end of the peptide in a separate step. For this purpose, FITC (3 equiv.) and DIEA (6 equiv.) were added to the peptidyl resin in the dark for 24 h. FITC-labeled peptide was cleaved from Rink Amide resin using the mixture cleavage solution TFA/TIS/H_2_O (95:2.5:2.5) for 2 h, precipitated with anhydrous, cold diethyl ether, and lyophilized. The obtained modified peptide was characterized using an analytical reverse-phase HPLC Shimadzu system (Prominence-i LC-2030C Plus, Shimadzu, Kyoto, Japan) with a Jupiter 4 µm Proteo, 90 Å, 4.6 × 250 mm column, with UV detection at λ = 224 nm, using the linear gradient method from 5 to 95% solvent B for 60 min at a flow rate of 1 mL/min., where solvent A was water and B was acetonitrile as eluents containing 0.1% TFA. ESI MS in positive ion mode (+) was performed using a single quadrupole mass spectrometer (LCMS 2020 Shimadzu, Japan). Isocratic elution, 60% B, where eluent A consisted of water and 0.1% formic acid (LCMS grade) and eluent B consisted of acetonitrile (LCMS grade) containing 0.1% formic acid as eluents containing 0.1% formic acid (FA), at a flow rate of 1.5 mL/min. FITC-labeled peptide characterization: yellowish solid; synthetic yield: 89%; HPLC purity > 90%; R_t_ 23.017 min.; ESI MS of peptide calculated value: 1253.43 (g/mol); observed value: [M+2H]^2+^ *m*/*z* = 628.3. 

### 2.3. General Fluorescence Measurements

We prepared all the metal ion solutions from Hg(NO_3_)_2_, Pb(NO_3_)_2_, MnCl_2_, ZnSO_4_, KCl, NaCl, Cd^2+^, and Cr^3+^ standard solutions in double-distilled water at a concentration of 10 mM. A stock solution of the peptide (10 mM) was prepared in double-distilled water and acetonitrile (9:1, *v*/*v*). Fluorescence spectra were measured using Multimode Microplate Reader Synergy H1MG (BioTek Instruments, Winooski, VT, USA) at an excitation wavelength of 480 nm on an F-bottom 96-well plate with a working volume per well of 200 µL (Greiner Bio-One International GmbH, Kremsmünster, Austria).

### 2.4. Fluorescence Response of FY7 Peptide for Hg^2+^ Ions: Metal Selectivity and Cross-Reactivity Studies

The fluorescent selection ability of FY7 to 8 metal ions (Hg^2+^, Pb^2+^, Mn^2+^, Zn^2+^, Cd^2+^, Cr^3+^, Na^+^, and K^+^) was investigated in MES buffer solutions (50 mM, pH 5.65). Separate solutions of each metal ion prepared at varying concentrations (0, 0.02, 0.05, 0.1, 0.5, 1, 2, 3, 5, and 10 µM) were individually added to the 5 μM FY7 solutions. The fluorescence spectra of the FY7 solutions with different concentrations of each metal ion were recorded.

A fluorescence titration experiment was used to investigate the binding properties and interactions of fluorescent molecules, such as FY7 with Hg^2+^ ions. For this purpose, the different concentrations of Hg^2+^ ion solutions (0, 0.02, 0.05, 0.1, 0.5, 1, 2, 3, 5, 10 µM) were added to the 5 μM FY7 in MES buffer solutions (50 mM, pH 5.65) and fluorescence spectra of the FY7 solutions were measured.

For the cross-reactivity studies, to ensure this specificity, it is essential to test the response of the FY7 peptide to its target ion, Hg^2+^, in the presence of other potentially interfering metal ions. The fluorescence responses of FY7 peptide (5 μM) to Hg^2+^ (0.5 equiv.) ions in the presence of seven different metal ions in MES buffer solutions (50 mM, pH 5.65) were measured both separately and in a mixture of all tested ions.

### 2.5. Characterization of the Peptide–Metal Ion Complexation

To determine the binding stoichiometry of FY7 peptide and Hg^2+^ ions, a Job’s plot was performed. Fluorescence emission intensity, which is the total concentration of FY7 (0–5 μM) and Hg^2+^ ions (5–0 μM), was used to probe the Job’s plot, with a constant concentration of 5 μM. The measurements were performed in MES buffer solutions with a pH of 5.65 and a concentration of 50 mM. The standard deviation was determined by repeating each proportional concentration three times.

We obtained the FTIR spectra of the free FY7 and FY7–Hg^2+^ complex in MES buffer solutions (50 mM, pD 5.65). The Invenio-R FTIR spectrometer (Bruker, Karlsruhe, Germany) was employed to measure the samples, which was equipped with an MTC detector that was cooled with liquid nitrogen. The cuvette was composed of two CaF_2_ windows that were separated by PTFE spacers with a thickness of approximately 90 μm. The temperature of 25 °C was maintained by mounting the cuvette within a thermostat housing. The spectra were acquired with a spectral resolution of 1 cm^−1^ and a total of 256 scans. The Savitzky–Golay method was employed to calculate the second derivatives, which involved 21 data points and a polynomial of degree 3. All solutions were prepared using heavy water (D_2_O). After 24 h of incubation, the FY7–Hg^2+^ complex, buffer components, and dry FY7 peptide were suspended in D_2_O and lyophilized. The spectrometer was continuously purified with dry nitrogen during the measurements to reduce the impact of water vapor on the peptide amide bands. The water vapor correction was conducted in accordance with the algorithm and protocol recommended by Bruździak [36]. The algorithm and measurement protocol implemented guaranteed the effortless and nearly complete elimination of water vapor interference.

### 2.6. Low-Volume Fluorescence Measurements

For low-volume measurements, a custom benchtop setup was designed. This setup included a λ_L_ = 487 nm laser source (Lambdawave, with built-in collimating lenses), an aspherical lens with a focal length *f* = 11 mm (C340TMD-A, Thorlabs Inc., Newton, NJ, USA) for focusing the beam on a sample container, and a pair of optical fibers equipped with a λ_F_ = 495 nm optical longpass filter. An optical detector (USB4000 spectrometer, Ocean Optics, Orlando, FL, USA) collected spectra in the 200–900 nm wavelength range. The setup was assembled on an optical breadboard and secured with stands, rails, posts, and a 3-axis micro-stage (Thorlabs Inc.) to ensure better alignment of the elements. The container used was a 15 mm long fused silica capillary with outer and inner diameters of 1000 and 500 μm, respectively. These capillaries accommodated a total volume of V_C_ = 2.9 μL of the sample and were filled via capillary action by simply submerging one end in the selected liquid.

Optical measurements were performed in dark conditions at a room temperature of approximately 21 °C. The USB4000 spectrometer was set to an integration time of 12 ms. The measurement and data processing procedure began with collecting a blank spectrum (F_B_) when the capillary was filled only with the buffer solution. Subsequently, after filling with a sample and exposing it to the laser beam, the emission fluorescence spectrum (F_E_) was registered. The difference between these two measurements (ΔF = F_E_ − F_B_) was used to identify the sample’s emission spectra. The USB4000 was set to average five repeatedly registered F_E_. The analysis involved tracing the peptide’s emission at λ_em_, specified as the fluorescence intensity at the emission maximum IF = ΔF(λ_em_).

## 3. Results and Discussion

In order to develop a peptide-based micro-volume glass capillary fluorometer for rapid and selective detection of mercury in aqueous samples, a metal-binding peptide identified through phage display biopanning [37] was used. The affinity of the FY7 sequence for heavy metal ions was tested.

### 3.1. FY7 Peptide Synthesis and Characterization

The metal ion-binding peptide was synthesized by Fmoc/tBu microwave-assisted solid-phase peptide synthesis [27].

FITC was introduced at the N–terminal side of the peptide in this study, preceded by a linker. In a microwave-assisted automated SPPS step, the β-Ala linker was incorporated into the growing peptide chain. The utilization of Fmoc-β-Ala-OH as a linker in SPPS is a strategic advantage in the synthesis of FITC-modified peptides, ensuring the production of functionally intact, high-purity FITC-labeled peptides that are suitable for a variety of applications. The separation of the bulky fluorophore from the bioactive peptide sequence and the prevention of side reactions during the TFA-mediated cleavage process in solid-phase peptide synthesis can be achieved by employing Fmoc-β-Ala-OH as a linker. The Fmoc-β-Ala-OH linker is a non-α amino acid, which means that it has a distinct structural configuration from standard α-amino acids. This distinction enables the spatial separation of the peptide chain from the fluorophore (FITC). Furthermore, the β-Ala linker can function as a protective spacer, thereby reducing the interaction between the reactive FITC group and the peptide backbone during the TFA cleavage process. This mitigates the likelihood of adverse reactions, including the unintended degradation of the modified peptide chain [38]. As a consequence, the fluorescent peptide was designed and produced with a high purity (>90%) and yield (89%) through TFA-mediated cleavage.

### 3.2. FY7 Peptide Metal Selectivity and Cross-Reactivity Studies

**Figure 1:**
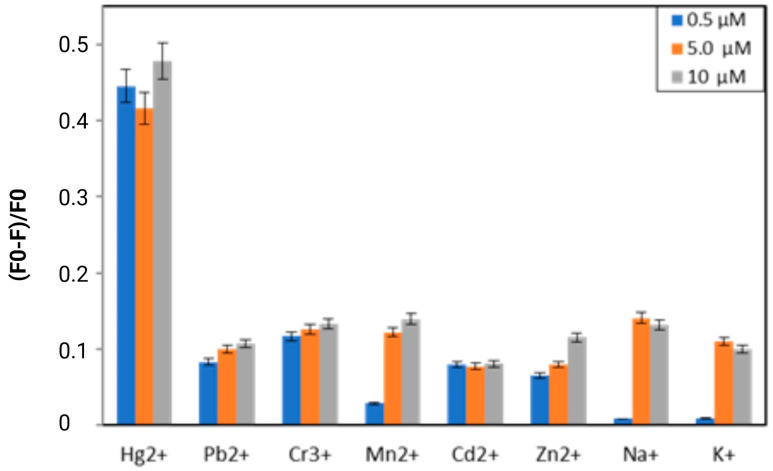
The selectivity of FY7 peptide (5 µM) for Hg^2+^ ions in MES buffer solutions (50 mM, pH 5.65). Concentrations of all metal ions were at 0.5, 5.0, and 10 µM. F0 and F were the fluorescence intensities of FY7 in the absence and presence of metal ions, respectively. Error bars represent the standard deviation of three independent measurements.

We conducted fluorescence measurements verified that the presented novel, FITC-labeled FY7 peptide can be employed as a biosensor that is both highly sensitive and specific to the Hg^2+^ ions. Initially, we investigated the affinity of the synthetic FY7 peptide for specific metal ions. In order to ascertain the selectivity of the FITC-labeled peptide (5 µM) toward eight distinct metal ions (Hg^2+^, Pb^2+^, Mn^2+^, Zn^2+^, Cd^2+^, Cr^3+^, Na^+^, and K^+^) in 50 mM MES buffer solutions at pH 5.65, the fluorescence spectra were analyzed. The FY7 probe exhibited a sensitive response exclusively to Hg^2+^ ions when compared to the fluorescence response of selected metal ions at three concentrations (0.5, 5.0, and 10 µM) (Figure 1). When the FY7 peptide was treated with the other seven metal ions, no such effect was observed, which was approximately 4.3 times less than in the case of Hg^2+^. The interaction with mercury ions resulted in a substantial reduction in fluorescence intensity, as evidenced by the fluorescence quenching rate of approximately 48% for Hg^2+^. FY7 demonstrated a high level of selectivity toward Hg^2+^ ions, as evidenced by the selectivity experiment results.

**Figure 2:**
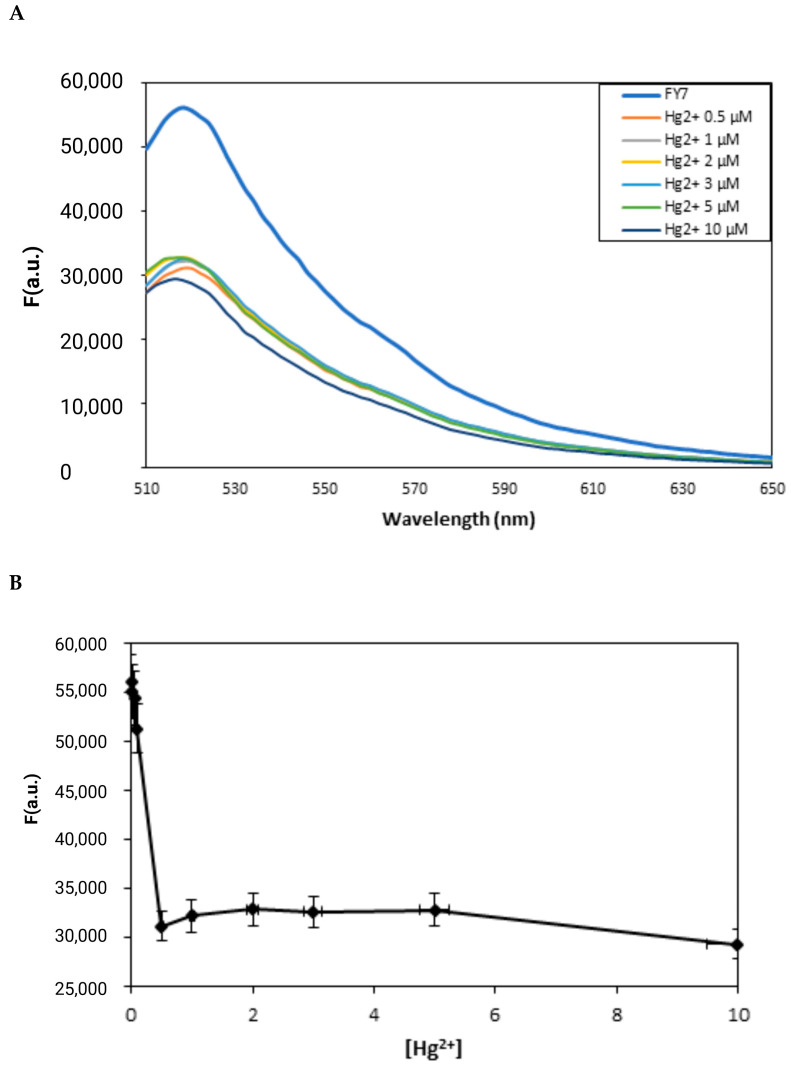
(**A**) Fluorescence emission spectra of FY7 (5 µM) upon the addition of Hg^2+^ ions (0–10 µM) in MES buffer solutions (50 mM, pH 5.65). (**B**) Plots of fluorescence intensity of FY7 as a function of Hg^2+^ ions concentration (µM). Error bars represent the standard deviation of three independent measurements.

Upon interaction with Hg^2+^, the fluorescence spectra of the FY7 peptide at varying concentrations of Hg^2+^ ions in MES buffer solution (pH 5.65) exhibited substantial changes (Figure 2). A prominent fluorescence peak at approximately 518 nm was observed in the free FITC-labeled peptide, FY7, in the absence of mercury (II) ions, as illustrated in Figure 2A. The fluorescence intensity of FY7 decreased significantly upon the addition of Hg^2+^ ions. The fluorescence titration spectra of FY7 (5 µM) with Hg^2+^ demonstrated that the fluorescence intensity of FY7 was significantly reduced when the peptide–Hg^2+^ complex was formed. Based on the turn-off response, Hg^2+^ is the sole metal ion that diminishes the fluorescence intensity of the FY7 sample. The interaction between the labeled peptide and Hg^2+^ ions was demonstrated by the decrease in fluorescence intensity. The photoinduced electron transfer (PET) effect resulted in a decrease in fluorescence intensity as a result of the complex formation between the FY7 peptide and Hg^2+^ ions. This phenomenon arises when the excited fluorophore (FITC) transfers an electron to the Hg^2+^ ion, which is a strong electron acceptor due to its high electron affinity. This process results in non-radiative relaxation and fluorescence quenching.

The addition of 0–0.5 µM Hg^2+^ ions to the peptide caused a gradual decrease in its fluorescence intensity (Figure 2A). Furthermore, a quenching rate of approximately 48% was observed at a higher concentration of Hg^2+^ (ranging from 0.5 to 10 µM) when a significant turn-off response was observed (Figure 2B). It is intriguing that the titration curve achieved a stable plateau with the addition of only 0.1 equivalent of Hg^2+^ ions.

The Hg^2+^ ion in water systems can be rapidly and consistently detected by the FY7 peptide. Furthermore, the fluorescent quenching effect (turn-off response) demonstrates that the FITC-labeled peptide possesses exceptional selectivity and sensitivity toward Hg^2+^ ions.

**Figure 3:**
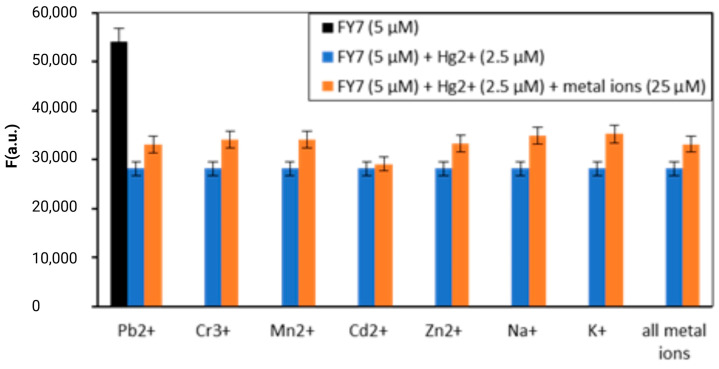
Fluorescence response of FY7 (5 µM) to Hg^2+^ (0.5 equiv.) in the presence of various metal ions (5 equiv.) in MES buffer solutions (50 mM, pH 5.65). Error bars represent the standard deviation of three independent measurements.

The fluorescence signal’s interpretation may be influenced by the peptide FY7’s response to multiple metal ions, which is referred to as cross-reactivity. In order to confirm the peptide biosensor’s affinity for mercury (II) ions, the FY7 probe was introduced into a mixture of Hg^2+^ ions (0.5 equiv.) and other 7 metal ions (5.0 equiv.) one at a time, and the resulting fluorescence emission spectra were analyzed. The fluorescence response of the FY7 was not affected by the presence of other divalent metal ions, such as Zn^2+^, Mn^2+^, and Cd^2+^, or trivalent chromium ions, as illustrated in Figure 3. Likewise, the presence of strong Lewis acid metal ions, such as Pb^2+^, did not interfere with the detection of Hg^2+^ ions, as they did not compete for binding to the peptide biosensor. Furthermore, the fluorescence emission of the peptide–Hg^2+^ complex was not significantly reduced by the simultaneous introduction of all the analyzed ions into the sample containing the FY7 peptide probe and Hg^2+^ ions. This property is essential for the development of selective biosensors that can accurately identify and measure specific metal ions in complex mixtures.

The results indicate that the fluorescence can be regulated by an ON–OFF switch, which is consistent with the FITC-labeled peptide biosensor’s potent chelating ability toward Hg^2+^ ions. It is imperative to emphasize that the FY7 peptide maintains a high level of responsiveness to Hg^2+^ ions, even in the presence of high concentrations of other competitive ions.

### 3.3. Molecular Characterisation of FY7 and Hg^2+^ Ions Interation

**Figure 4:**
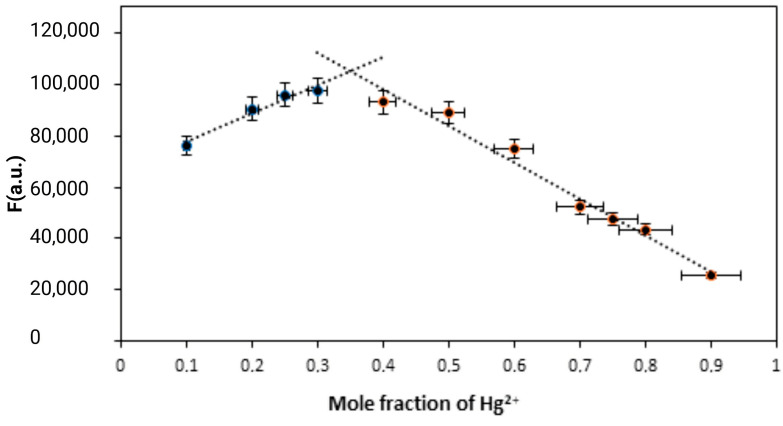
Job’s plot for determining the stoichiometry of FY7 and Hg^2+^ in MES buffer solutions (50 mM, pH 5.65), the total concentration of FY7 and Hg^2+^ was 5 µM. Error bars represent the standard deviation of three independent measurements.

The stoichiometry of a complex formed between two interacting species, such as a peptide and metal ions, can be determined using the Job’s plot, which is also referred to as the continuous variation method. It encompasses the preparation of a sequence of solutions in which the mole fractions of the two reactants (in this instance, the FY7 peptide and Hg^2+^ ions) are varied, while the total molar concentration of the two reactants remains constant. The mole fraction of one of the components is plotted against the fluorescence response. The stoichiometry of the complex is determined by the point at which the response is at its maximum. A Job’s plot experiment was conducted to determine the stoichiometry of the interaction between the FY7 peptide and Hg^2+^ in MES buffer (50 mM, pH 5.65). The Job’s plot of fluorescence intensity, which is influenced by the complex formation, was plotted as a function of the mole fractions of Hg^2+^ (Figure 4). The stoichiometry of the complex formed between the FY7 peptide and Hg^2+^ ion was determined to be 2:1 based on the Job’s plot, which shows the maximum fluorescence intensity at a mole fraction of 0.35 for Hg^2+^. This implies that one Hg^2+^ ion is bound by two FY7 peptides.

**Figure 5:**
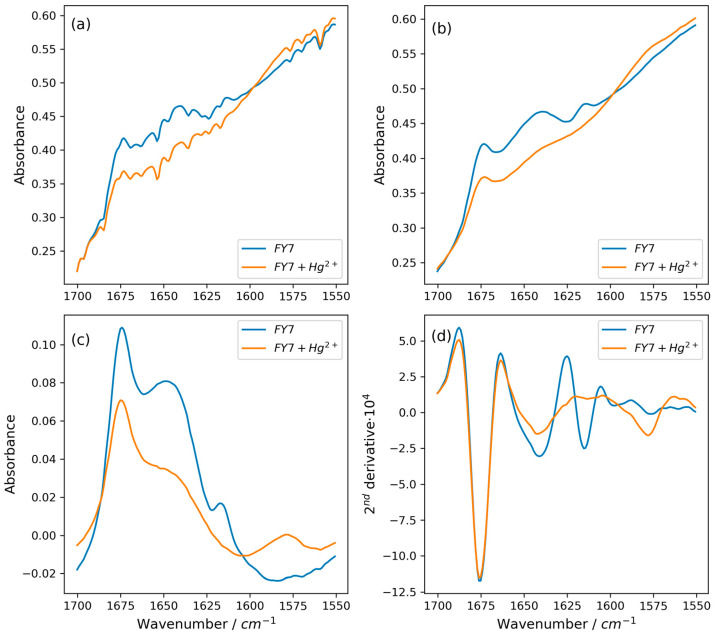
(**a**) Raw spectra of FY7 peptide (blue line) and FY7–Hg^2+^ complex (orange line) in the amide bands region of the peptide. (**b**) Spectra after water vapor correction. (**c**) Spectra after removing the contribution of D_2_O in the studied range. (**d**) Second derivatives of FTIR spectra. Please see a description in the text.

FTIR was used to verify that a complex had formed between the peptide and Hg^2+^ ions. In order to determine which chemical groups of the FY7 peptide are crucial for the interaction with Hg^2+^ (Figure 5), an FTIR experiment was conducted. FTIR can be used to obtain information regarding the molecular vibrations and structures of the peptide and functional groups. D_2_O, as a solvent, enables the identification of amide bands in the peptide, which are directly associated with potential structural modifications that may result from interactions with mercury ions. The amide band region of the spectra of the peptide and peptide–metal complexes was virtually unaffected by the buffer solution, which contained acetonitrile, salts, and buffer components. The asymmetric stretching of the carboxyl group of trifluoroacetate (TFA) in D_2_O is responsible for the strong peak at 1675 cm^−1^ [39,40]. The presence of this band is readily apparent in spectra that contain peptides. We opted not to subtract it, as it could introduce subtraction artifacts that are easily misinterpreted. Nevertheless, we calculated the second spectra derivatives to improve the sub-band changes in the complex amide I’ band. The presence of residual TFA does not significantly affect the analysis of IR spectra, as the position of the associated band is clearly defined, readily identifiable, and remains consistent without observable shifts or alterations across all spectra. This may suggest that it does not interact significantly with the solution components.

The FTIR spectrum of free FY7 (blue line) is significantly different from that of FY7 saturated with Hg^2+^ ions (orange line) (Figure 5d). The primary backbone band (1670–1625 cm^−1^) expands, but its overall shape does not suggest the formation of any new secondary structures, such as aggregates or intermolecular β-sheets [41]. Nevertheless, the binding of Hg^2+^ induces a shift in the clearly separated bands that correspond to amino acid side chains. The v(CC) and in-plane δ(CH) vibrations of the tyrosine ring are responsible for a signal at approximately 1615 cm^−1^ in the free FY7 peptide. Upon the formation of the complex, the intensity of this band experienced a substantial decrease and may have shifted to approximately 1575 cm^−1^. This suggests that the ring or its fragment was directly involved in the binding interaction with the metal ions. The Hg^2+^ ion induces the formation of a new coordination environment within the peptide, with the tyrosine residue being the primary component. In conclusion, the ion exerts a significant influence on the Tyr residue of the peptide while not affecting its secondary structure in solution. Such a change may indicate a direct Hg^2+^–Tyr interaction; however, without other structural evidence, the exact mechanism of such a binding is a matter of debate.

### 3.4. Low-Volume Miniaturized Optical Nano-Biosensor for the Rapid, Sensitive, and Selective Detection of Toxic Mercury in Aqueous Samples Based on FY7 Peptide

**Figure 6:**
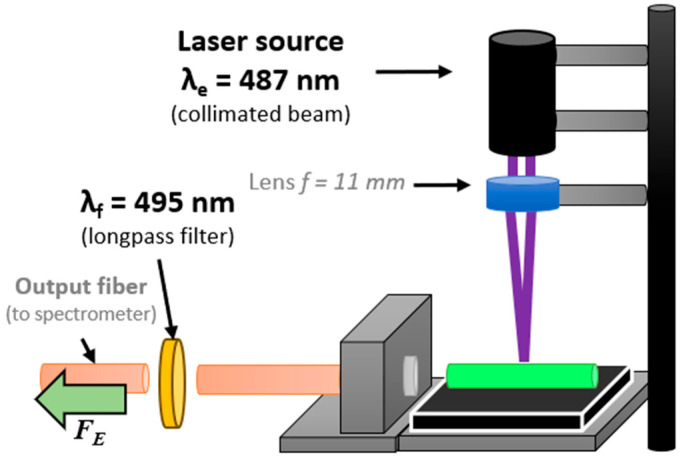
Schematic representation of an experimental setup for micro-volume capillary measurements.

**Figure 7:**
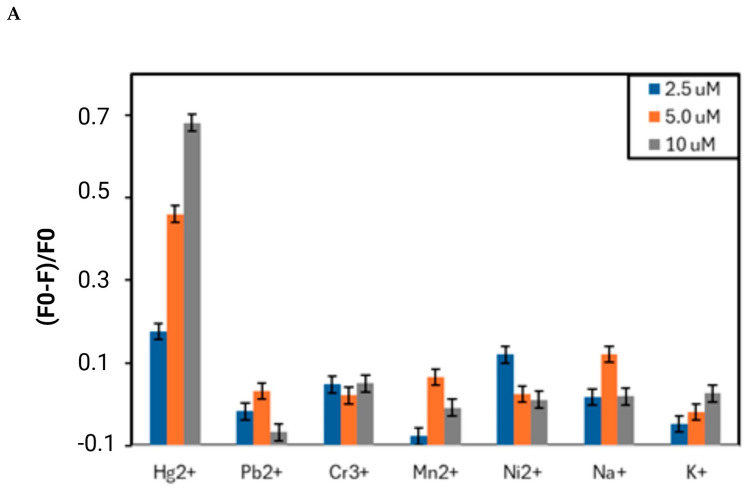

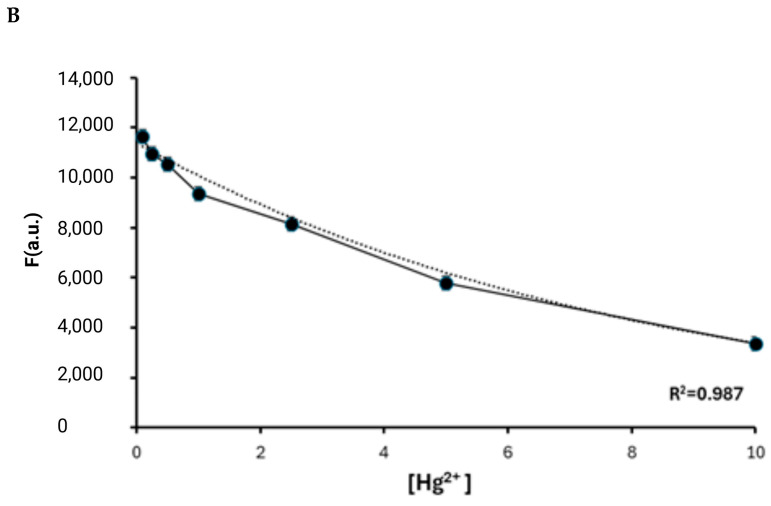
(**A**) The selectivity of the FY7 peptide (50 µM) for Hg^2+^ ions (2.5 µM) in MES buffer solutions (50 mM, pH 5.65) measured in a low-volume fluorescence setup. Concentrations of all metal ions were at 0.5, 5.0, and 10 µM. F0 and F were the fluorescence intensities of FY7 in the absence and presence of metal ions, respectively. (**B**) Plot of fluorescence intensity of FY7 as a function of Hg^2+^ ion concentration (µM); the dotted line is an approximation used for LOD estimation.

The fluorescence response of FY7 peptide was measured in the custom, low-cost glass capillary setup. The results achieved for the H1MG spectrofluorometer were considered a reference and used as a benchmark for this custom, miniaturized setup (Figure 6). As a first step, similarly as for H1MG, FY7 selectivity toward distinct metal ions was assessed. After initial tests, the peptide concentration, when using the miniaturized setup, was increased to 50 µM (from 5 µM in H1MG) in order to achieve a more robust dynamic response within the limited parameters of the small USB4000 spectrometer. Similar to prior tests, the FY7 peptide in capillaries was analyzed in a 50 mM MES buffer solution at pH 5.65, but with a modified set of seven distinct metal ions (Hg^2+^, Pb^2+^, Mn^2+^, Ni^2+^, Cr^3+^, Na^+^, and K^+^). The commercial setup utilized ion solutions that were in the 0.02–10 µM range, while in the low-cost setup, the concentrations tested were 0.1, 0.25, 0.5, 1, 2.5, 5, and 10 µM. With the inclusion of nickel, six of the metal ions that were chosen remained unchanged from the H1MG commercial setup. Despite the lower dynamic range of the low-cost setup, the FY7 response for distinct metal ions matched the results from H1MG (Figure 7A). Based on fluorescence intensity changes in the presence of Hg^2+^ ions, an approximation was made to identify the theoretical limit of detection (LOD). The LOD was determined for 3σ while the approximation curve approached maximum fluorescence intensity of pure FY7, where measurement uncertainty was σ = 1.84% for N = 20 sample size (Figure 7B). The identified theoretical LOD for Hg^2+^ detection was 0.02 µM.

**Figure 8:**
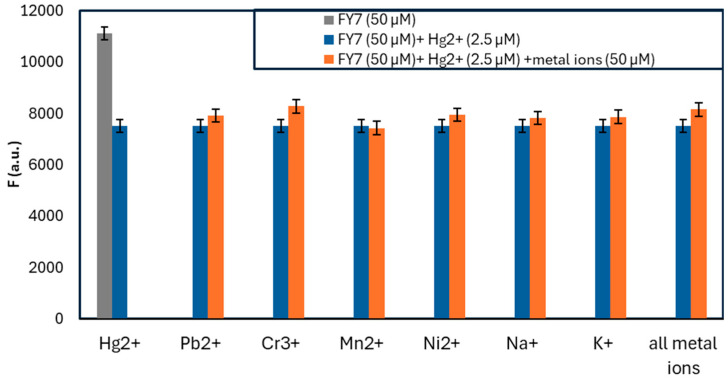
Fluorescence response of FY7 (50 µM) to Hg^2+^ (2.5 µM) in the presence of various metal ions (10 µM) in MES buffer solutions (50 mM, pH 5.65) measured in a low-volume fluorescence setup.

The response of the FY7 peptide to the analyte in the presence of interfering metal ions, also referred to as cross-reactivity, may influence the obtained fluorescence signal. Therefore, the FY7 probe (50 µM) was placed in a solution of Hg^2+^ ions (2.5 µM) and other 7 metal ions (50 µM) respectively, and the ensuing fluorescence emission spectra were examined (Figure 8).

The obtained results with the low-volume setup were in accordance with the commercial setup, as the FY7 peptide demonstrated a sensitive response exclusively to Hg^2^^+^ ions. The observed fluorescence emission was slightly higher, up to 65% in the presence of Hg^2^^+^ ions. For other metal ions, FY7’s measured intensity fluctuated by approximately 3σ, but with no observable trend, closely matching the results from H1MG. Both tests demonstrated the robustness of the FY7 peptide as well as confirmed the potential of the low-volume measurements, as the sample volume used per one measurement was 69 times lower. The miniaturized capillary setup was verified as a suitable alternative to commercial devices, and in addition, it offers flexibility to be used as a fully mobile POC (point-of-care) device.

## 4. Conclusions

There are numerous chromatographic and spectroscopic methods available for the detection of toxic mercury (Hg^2+^) in water; however, there is still a need for simple, rapid, inexpensive, and sensitive strategies. Only a few descriptions have been provided thus far, and they are predicated on the colorimetric and fluorescence detection of Hg^2+^ in water. The literature describes a straightforward procedure that is based on the lysine-induced aggregation of citrate-capped gold nanoparticles (AuNPs) in the presence of Hg^2+^ ions with a limit of detection (LOD) of 2 nM [42]. Other colorimetric sensor studies that have been described are based on green synthesized silver chloride nanoparticles (Ag@AgCl-NPs), which have demonstrated an intriguing property for the detection of hazardous Hg^2+^ in water. The colorimetric assay is predicated on the concentration-dependent degradation of as-prepared Ag@AgCl-NPs in the presence of Hg^2+^. The detection limit of this cost-effective assay is 4.19 nM, which is below the threshold established by the Chinese agency and, more importantly, below the threshold established by the U.S. Environmental Protection Agency for potable water [43]. Additionally, the citrate-capped AuNPs exhibit a highly selective colorimetric response to Hg^2+^ in the presence of lysine [44]. Furthermore, an example of fluorescent gold nanoprobes has been described [45]. Finally, a FY7 phage display-derived peptide was employed for Hg^2+^ ion detection using a low-cost, low-volume capillary setup. With sample volume of only 2.9 µL per measurement and simple optical equipment, the FY7 peptide produced comparable results to those obtained from a professional spectrofluorometer. The estimated detection limit of FY7 for Hg^2+^ was 0.02 µM. The WHO’s recommended limit of 0.03 µM Hg^2+^ in drinking water is in line with the detection limit for low-volume measurements. The results in the presence of other metal ions confirmed FY7’s robustness, specificity, and high proficiency in conformational changes. Notably, the combination of the low-cost capillary system with the synthesized, efficient FY7 peptide presents a cost-effective and strong alternative to conventional commercial spectrofluorometers. This straightforward setup is affordable, easy to use, highly sensitive, and well-suited for portable measurements. The FY7 peptide, when used with such system, offers flexibility and is suitable for a wide range of applications, including environmental monitoring of mercury (II) ions.

## 5. Patents

The results of this work were included in the patent application number PL 449082 entitled “Synthetic 7-amino acid peptide linked by a beta-alanine linker with a FITC fluorophore as a nano-biosensor for the detection of toxic mercury (II) ions”.

## Data Availability

All research data will be made available upon request.
